# Insights into the Binding Mode of Lipid A to the Anti-lipopolysaccharide Factor ALFPm3 from *Penaeus monodon*: An In Silico Study through MD Simulations

**DOI:** 10.1021/acs.jcim.3c00173

**Published:** 2023-04-07

**Authors:** Cristina González-Fernández, Christoph Öhlknecht, Matthias Diem, Yerko Escalona, Eugenio Bringas, Gabriel Moncalián, Chris Oostenbrink, Inmaculada Ortiz

**Affiliations:** † Departamento de Ingenierías Química y Biomolecular, Universidad de Cantabria, Avda. Los Castros, s/n, 39005 Santander, Spain; ‡ Institute for Molecular Modeling and Simulation, BOKU-University of Natural Resources and Life Sciences, Muthgasse 18, 1190 Vienna, Austria; § Departamento de Biología Molecular, Universidad de Cantabria and Instituto de Biomedicina y Biotecnología de Cantabria (IBBTEC), Universidad de Cantabria-CSIC, 39011 Santander, Spain

## Abstract

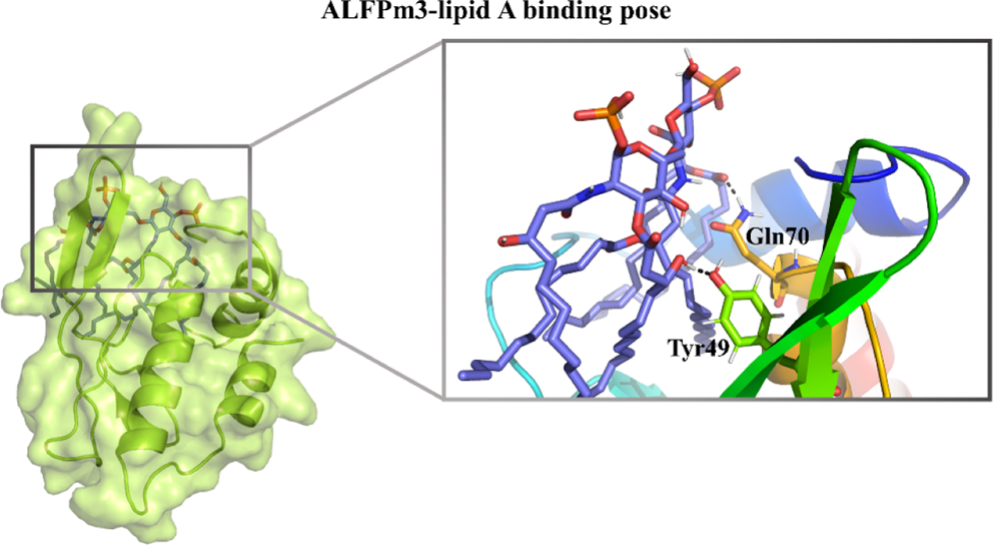

The globally expanding threat of antibiotic resistance calls for the development of new strategies for abating Gram-negative bacterial infections. The use of extracorporeal blood cleansing devices with affinity sorbents to selectively capture bacterial lipopolysaccharide (LPS), which is the major constituent of Gram-negative bacterial outer membranes and the responsible agent for eliciting an exacerbated innate immune response in the host during infection, has received outstanding interest. For that purpose, molecules that bind tightly to LPS are required to functionalize the affinity sorbents. Particularly, anti-LPS factors (ALFs) are promising LPS-sequestrating molecules. Hence, in this work, molecular dynamics (MD) simulations are used to investigate the interaction mechanism and binding pose of the ALF isoform 3 from *Penaeus monodon* (ALFPm3), which is referred to as “AL3” for the sake of simplicity, and lipid A (LA, the component of LPS that represents its endotoxic principle). We concluded that hydrophobic interactions are responsible for AL3–LA binding and that LA binds to AL3 within the protein cavity, where it buries its aliphatic tails, whereas the negatively charged phosphate groups are exposed to the medium. AL3 residues that are key for its interaction with LA were identified, and their conservation in other ALFs (specifically Lys39 and Tyr49) was also analyzed. Additionally, based on the MD-derived results, we provide a picture of the possible AL3–LA interaction mechanism. Finally, an in vitro validation of the in silico predictions was performed. Overall, the insights gained from this work can guide the design of novel therapeutics for treating sepsis, since they may be significantly valuable for designing LPS-sequestrating molecules that could functionalize affinity sorbents to be used for extracorporeal blood detoxification.

## 1 Introduction

Lipopolysaccharide (LPS), also known as endotoxin, is the major constituent of Gram-negative bacterial outer membranes and often has crucial implications in bacterial pathogenicity.^1−6^ LPS is an amphiphilic molecule and possesses a tripartite structure that consists of the lipid A (LA), the core oligosaccharide, and the O-antigen.^4,7−9^ LA is made up of a β-(1 → 6)-linked glucosamine disaccharide backbone that is typically phosphorylated and acylated with a number of acyl chains that ranges from four to eight.^8,10−12^ The LA moiety, which is the most conserved component of LPS, is the endotoxic principle of LPS and acts as a pathogen-associated molecular pattern.^3,5,8,13^ Thereby, upon bacterial infection, LA is recognized by the host through the pattern recognition receptor toll-like receptor 4/myeloid differentiation factor 2 (TLR4/MD2) complex, which results in the activation of the innate immune response in order to accomplish the clearance of the bacterial infection.^11,13−16^ A balanced host response is vital in order to prove advantageous for eliminating bacteria; conversely, an exaggerated immune response can lead to sepsis, which is a life-threatening condition with tremendous morbidity and mortality globally.^11,14,17−21^

To abate the expected increasing trend of antibiotic resistance, and consequently of sepsis, developing novel strategies for treating Gram-negative bacterial infections is an urgent need.^22−24^ In this regard, the extracorporeal removal of endotoxins from blood has been understood as a promising strategy.^25,26^ Particularly, the design of detoxification systems based on affinity sorbents, which rely on the immobilization of molecules that exhibit high affinity to LPS, has been the focus of intense research.^18,27−29^ Therefore, the selection of an appropriate molecule to functionalize the affinity sorbents is crucial for their successful implementation for LPS sequestration. Several molecules, either synthetic or natural, have been reported to interact with LPS.^23,27,28,30^ Particularly, anti-LPS factors (ALFs), which are antimicrobial peptides identified in marine chelicerates and crustaceans, have been recognized as potential LPS-sequestrating molecules due to their avid binding to endotoxins.^31−33^ Elucidating the interaction mechanism of ALFs and LPS, as well as the LPS binding site in ALFs, will be valuable to go further in the design of LPS-sequestrating molecules to be anchored on affinity sorbents for detoxification purposes. Therefore, the identification of the LPS binding site of both the horseshoe crab *Limulus polyphemus* ALF (LALF) and the ALF isoform 3 from shrimp *Penaeus monodon* (ALFPm3), which will be referred to as “AL3” for the sake of simplicity, has received significant interest.^31,34^ For example, Hoess et al.^34^ determined the crystal structure of a recombinant LALF and suggested that the LPS binding site in LALF probably entails an amphipathic loop. They proposed that lipopolysaccharide-binding protein (LBP) and bactericidal/permeability-increasing protein (BPI), which are mammalian proteins, also share this LPS binding motif. Similarly, Yang and co-workers^31^ determined the three-dimensional (3D) structure of recombinant AL3 by nuclear magnetic resonance (NMR), which is almost identical to that of LALF (see Section S1 of the Supporting Information), and they tried to study experimentally the interaction of this protein with LPS, LA, and a LA analogue. However, the insolubility of LA in water and the large molecular size of the AL3–LPS and AL3–LA analogue complexes hampered the experimental determination of the 3D structure of AL3 in complex with the LA derivatives by standard NMR techniques, and thus the elucidation of their binding site in AL3. Therefore, they mapped a putative binding site by performing a structural comparison of the AL3 structure with that of FhuA (outer membrane protein of *Escherichia coli* that transports the ferric siderophore ferrichrome and also acts as a receptor for phages)^35−37^ in the FhuA–LPS complex on the basis of the hypothesis they proposed. Such hypothesis establishes that a similar LA binding site is shared by LPS-binding proteins. From this approach, Yang et al.^31^ proposed several amino acids that could belong to the LA binding site and suggested that the binding pose may involve the surrounding of the AL3 structure by the LA acyl chains.

Although the works of Hoess et al.^34^ and Yang et al.^31^ have reported interesting findings, the LPS/LA binding sites they hypothesized have not been demonstrated yet. Additionally, to the best of our knowledge, there are no further studies that either in silico or experimentally prove the LPS/LA binding site of ALFs or their interaction mechanism. However, understanding how ALFs and LPS interact and recognizing the LPS/LA binding site of ALFs are key for progressing in the design of LPS-sequestrating molecules, since modifications to the ALFs structure could be introduced to enhance the strength and specificity of their interaction with the endotoxin.

In this work, we gain insights into the interaction of AL3 and *E. coli* LA through molecular dynamics (MD) simulations, thus contributing to progress in the design of LPS-sequestrating molecules. More specifically, we elucidate an AL3–LA binding pose and, hence, delineate the LA binding site of AL3. Additionally, amino acids that are key for AL3–LA recognition and their stable binding have been identified, and an energetic characterization of the AL3–LA interactions to thermodynamically demonstrate the nature of their binding has been performed. We emphasize the AL3 conformational changes upon LA binding and demonstrate the reversibility of the AL3–LA binding despite such conformational changes. On the basis of the in silico findings, we propose a possible interaction mechanism between AL3 and LA. Furthermore, we demonstrate the conserved character of the AL3 amino acids we identified to be crucial for the interaction with LA in LALF. Finally, the in vitro validation of in silico predictions has been addressed through site-directed mutagenesis (SDM) and binding tests. Collectively, the knowledge gained from this study paves the way for the rational design of LPS-sequestrating molecules to be anchored on affinity sorbents for extracorporeal blood detoxification. Hence, this work contributes to the design of novel therapeutics for treating sepsis.

## 2 Results and Discussion

Shedding light on the interaction mechanism and binding mode of AL3 and LPS could prove valuable for the design of novel molecules to be used for LPS sequestration. Here, the LA portion, instead of the whole LPS molecule, has been considered for the MD simulations. This choice, which considerably reduces the complexity of the system, arises from the following observations: (i) LA plays a crucial role in the development of sepsis since it harbors the endotoxic properties of LPS, and (ii) the LA moiety of LPS is key for several LPS-molecule binding events.^8,38−40^ Thereby, in this work, we provide insights into the AL3–LA interaction mechanism and binding mode through MD simulations and also address the experimental validation of these findings.

### 2.1 Elucidation of the 3D Structure of the AL3–LA Complex

**Figure 1:**
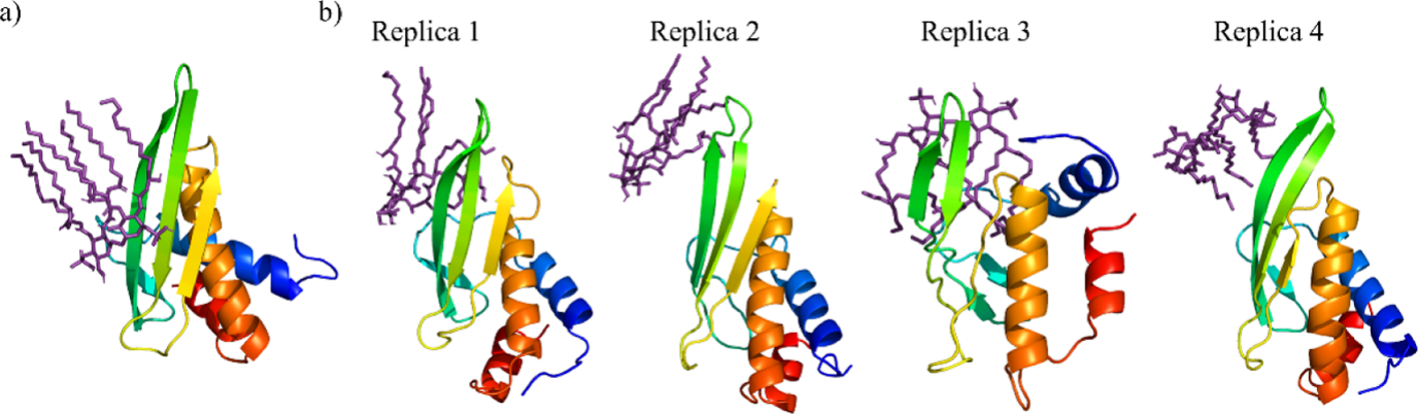
(a) Initial AL3–LA complex structure for the simulations, (b) snapshots of the AL3–LA complex at the end of the four MD simulations. AL3 is rainbow-colored from the N-terminus (blue) to the C-terminus (red).

Insights into the AL3–LA interaction mechanism and binding mode were gained by performing MD simulations of LA bound to AL3 in a 150 mM NaCl buffer. The initial complex structure for these simulations was derived following a similar procedure to that used by Yang et al.^31^ to hypothesize the LA binding site of AL3, as has been detailed in the Methods section of the Supporting Information. In such initial complex structure, LA spreads out over the external side of the protein β-hairpin, with the phosphates oriented downward and the acyl chains upward, as can be seen in Figure 1a. However, snapshots taken at the end of the four MD simulations of the AL3–LA complex (Figure 1b) reveal that when both the protein and the lipid are allowed to freely move and interact, the lipid leaves the external side of the protein β-hairpin and tends to reach the protein cavity (PC). This tendency is observed in all four simulations (as corroborated by the clustering analysis in Figure S4); however, the complete insertion of LA in the AL3 cavity, where it is oriented upward with the phosphates exposed to the medium and the acyl tails buried in the protein cleft, was only attained in the third replica. The fact that in all simulations the lipid tries to reach the protein cleft, along with the high hydrophobic character of the lipid (see Section S2 of the Supporting Information), supports the location of the LA binding site in the PC as well as the abovementioned LA orientation in it. It is worth mentioning that this AL3–LA binding mode unveiled from MD simulations resembles that of LA with MD2, which has been experimentally determined through X-ray crystallography (Protein Data Bank, PDB, ID: 3FXI). Additionally, the fact that the insertion of LA in the PC is not always achieved was also discussed by Garate and Oostenbrink.^10^ Particularly, they performed three MD simulations of MD2 with LA located outside the PC, and only in one of them was the lipid able to insert itself in such cavity; thereby, they revealed that there is a competition between the MD2-LA binding and the closing of the MD2 cavity. Thus, the cavity opening could be understood as the bottleneck for achieving the lipid burial in the AL3 and MD2 clefts.

Collectively, the abovementioned findings, namely, (i) the LA binding in the AL3 cleft with the phosphates exposed to the medium and its aliphatic tails buried in the PC, and (ii) the difficulty of LA insertion in the protein cleft due to the closure of the PC, are consistent with the MD2-LA crystallographic structure^41^ and the work of Garate and Oostenbrink.^10^ Therefore, our in silico-derived insights are supported by previously reported works.

**Figure 2:**
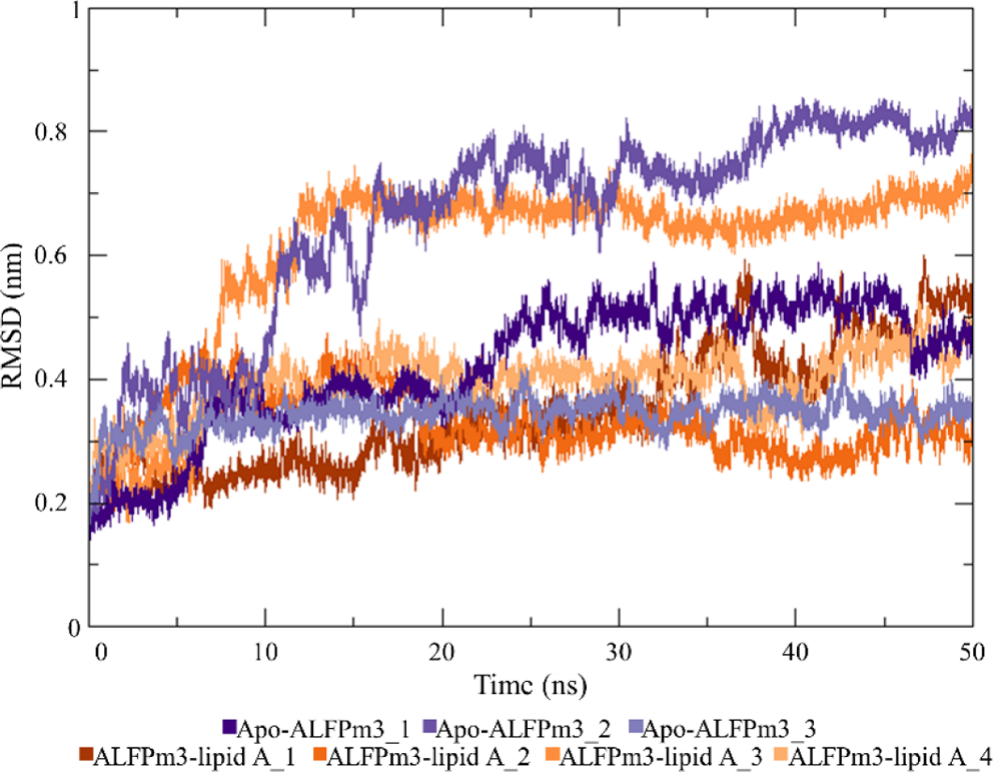
RMSD of AL3 backbone atoms in the apo and bound states.

The LA binding in the AL3 cavity requires the opening of such cavity, and thus conformational changes on the protein, so that it could accommodate the lipid. Hence, the atom-positional root-mean-square deviation (RMSD) of the protein backbone atoms (C-α, N, and C) with respect to the AL3-minimized structure was derived. It is compared to the values obtained from the simulations of the apo-AL3 in Figure 2. For most of the simulations, the RMSD value remained below 0.6 nm, except for replica 2 of the apo simulations and replica 3 of the simulations with LA bound. This shows that in order to fully accommodate the LA tails in the hydrophobic core of the protein, a significant change is required and that similarly sized structural changes are possible as well in the apo state of the protein. This suggests that the binding of LA could indeed involve an induced fit or conformational selection model.

**Figure 3:**
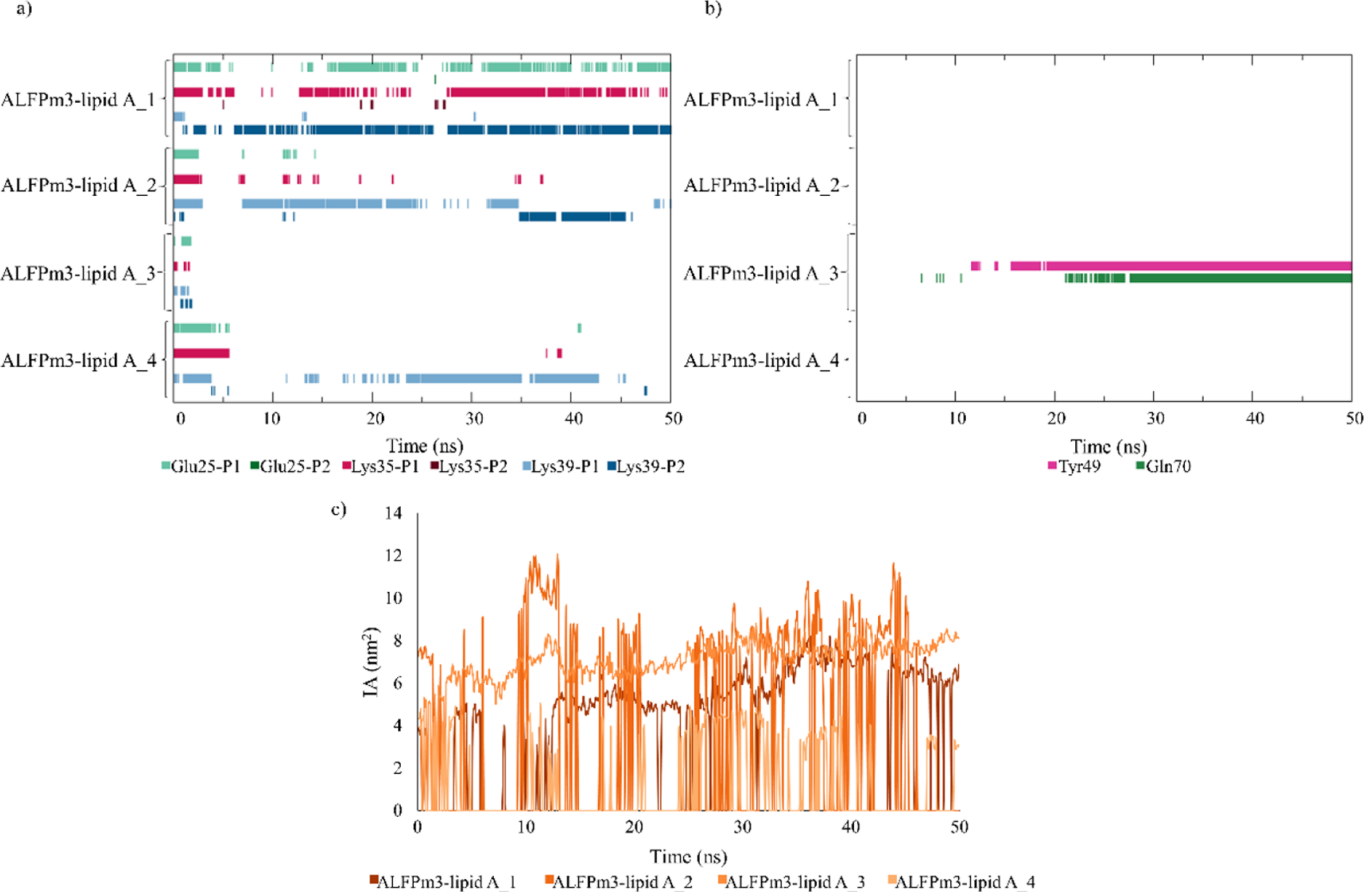
Occurrence of (a) salt bridges and (b) hydrogen bonds between AL3 and LA; (c) IA of the AL3–LA complex.

To gain further insight into the AL3–LA interaction mechanism and the binding mode, we determined the AL3 residues involved in the interaction with LA. For that purpose, the salt bridges and hydrogen bonds that are formed between these molecules were computed. According to Figure 3a,b, when the lipid reaches the PC (third replica), stable salt bridges along the simulation are not observed, but these molecules bind through stable hydrogen bonds. It is worth mentioning that in this work a hydrogen bond is considered to be long-lived when its percentage of occurrence over the trajectory is higher than 40%. Specifically, Tyr49 and Gln70, which are located in the PC, establish highly occurring hydrogen bonds with the lipid (percentage of occurrence of 68.10 and 44.07% for Tyr49 and Gln70, respectively). Interestingly, these hydrogen bonds are not initially present, but they appear as the lipid is inserted in the AL3 cleft. Conversely, when the lipid is not able to entirely reach the protein cleft (first, second, and fourth replicas), it establishes long-lived salt bridges with AL3 residues exposed to the medium, namely Glu25, Lys35, and Lys39. Yang et al.^31^ also reported the interaction of these amino acids (i.e., Lys35, Glu25, and Lys39) with LA. In detail, the P1 phosphate of LA (i.e., the phosphate located at position 4′ of the glucosamine GlcN II, see Figure S2) forms salt bridges with Lys35, Glu25, and Lys39, whereas the P2 phosphate (i.e., the one located at position 1 of the glucosamine GlcN I, see Figure S2) only interacts on a relatively regular basis with Lys39. Furthermore, the salt bridges that involve Lys39 are highly stable along the simulation in all the replicas where the lipid does not reach the PC. However, in these simulations, short-lived hydrogen bonds (percentage of occurrence lower than 40%) are observed and/or they do not involve amino acids located in the PC (such as Tyr49 or Gln70); for these reasons, the hydrogen bonds that occur in the first, second, and fourth replicas are not shown in Figure 3b. Hence, for these simulations, AL3 and LA mainly bind through salt bridges.

Furthermore, we computed the interface area (IA) of the contact between AL3 and LA to assess the stability of their binding. More specifically, an IA oscillating around a constant value, which implies that these molecules are bound throughout the entire simulation, represents a stable binding. On the other hand, when the binding is unstable, the lipid and the protein are not constantly in contact, but at certain times water molecules and/or ions (Na^+^ and/or Cl^–^) move in between them, which causes the IA value to drop to zero. According to Figure 3c, an IA oscillating around the same value (∼7 nm^2^), that is, a stable binding, is only obtained for the third replica; it is worth noticing that in this simulation the lipid acyl chains are tethered toward the PC. The other replicas, where the lipid does not completely reach the protein cleft, exhibit an unstable binding, as noticed from the drop to zero of the IA value. The fact that a stable IA is derived when the lipid is fully inserted in the PC also endorses the location of the LA binding site in the cleft of AL3 and the orientation of the lipid in such binding site with the phosphates exposed to the medium and the acyl chains buried in the cavity.

Finally, the nature and strength of the AL3–LA binding were assessed by computing the free energy of binding using the linear interaction energy (LIE) method (see the Methods section of the Supporting Information); these calculations are presented in Table 1. According to the binding free energy (Δ*G*_bind_) values, AL3 and LA bind more favorably in the third replica, that is, when the lipid is inserted in the PC. The electrostatic (Δ*G*_bind_^elec^) and van der Waals (Δ*G*_bind_^vdw^) contributions to the binding free energy demonstrate that hydrophobic interactions between the acyl chains of LA and the hydrophobic cavity of AL3 dominate the binding. This conclusion comes from the more favorable van der Waals component of the binding free energy in comparison to the electrostatic one. Thus, since AL3–LA binding is mainly driven by hydrophobic interactions, it would be expected that the LA binding site of AL3 is located in its cleft, as such cavity comprises hydrophobic amino acids. On the other hand, the Δ*G*_bind_ values lead to binding constants for the first, second, and fourth simulations that are 2 or 3 orders of magnitude lower than that for the third replica, which denotes that the strongest AL3–LA binding is observed for the third replica (*K*_bind_ = 2 × 10^4^ M^–1^). This binding constant is similar to that for the binding of LPS with several biomolecules, as reported by Basauri et al.^30^

**Table 1:** AL3–LA Binding Free Energies

	Replica 1	Replica 2	Replica 3	Replica 4
Δ*G*_bind_^vdw^ (kJ·mol^–1^)	–21 ± 3	–11 ± 1	–21 ± 1	–10 ± 1
Δ*G*_bind_^elec^ (kJ·mol^–1^)	6 ± 2	–1 ± 1	–3 ± 1	1 ± 1
Δ*G*_bind_ (kJ·mol^–1^)	–15 ± 3	–12 ± 1	–24 ± 1	–9 ± 1

Collectively, gathering the high hydrophobic content of LA, the outcomes of the simulations, and the previously reported results about the MD2-LA binding pose^10^, it is reasonable to expect that the lipid tries to bury itself in the PC, where hydrophobic amino acids are located. However, this binding pose differs from the one proposed by Yang and coworkers,^31^ who suggested that the lipid aliphatic tails might surround AL3.

The location of the LA binding site in the PC entails the fulfillment of two requirements. On the one hand, a stable binding in the protein cleft implies that the lipid remains in the PC during the simulations; otherwise, the LA binding site would not be located in the PC as the lipid tries to search for another binding site more energetically comfortable. On the other hand, the LA binding in the AL3 cleft calls for the recovery of the initial protein structure upon lipid removal from the cavity due to the reversibility of the AL3–LA binding despite its conformational changes for opening the cavity to accommodate the lipid. In this regard, the stability of the LA binding in the protein cleft and the reversibility of the AL3–LA binding have been examined in the following subsection.

### 2.2 Assessing the LA Binding Site of AL3

First, simulations of the AL3–LA complex in a 150 mM NaCl buffer using as the initial structure one where the lipid is inserted on the cleft of AL3 were performed in order to confirm that the LA-binding site of AL3 is located in the protein cleft. Specifically, such initial structure corresponds to the one from the third replica of the previous set of simulations with the most favorable van der Waals interaction energy since, as previously discussed, the AL3–LA binding is mainly driven by hydrophobic interactions. For the sake of clarity, from now on, we will refer to the simulations detailed in the previous section as “AL3–LA simulations”, and to the simulations where the initial complex structure was derived from the “AL3–LA simulations” as “AL3–buried LA simulations” (see Table S1 for further details about the simulations’ nomenclature).

**Figure 4:**
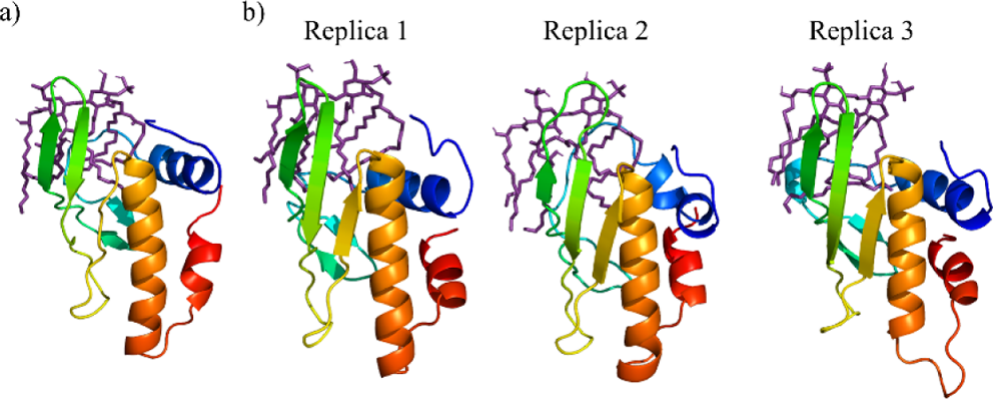
(a) Initial AL3–LA complex structure for the AL3–buried LA simulations and (b) snapshots of the AL3–LA complex at the end of the AL3–buried LA simulations. AL3 is rainbow-colored from the N-terminus (blue) to the C-terminus (red).

In Figure 4, the initial AL3–LA complex structure used for the AL3–buried LA simulations and a snapshot taken at the end of these simulations have been depicted. It can be perceived that the lipid remains tethered inside the PC at the end of the three replicas and that significant conformational changes of the AL3–LA complex from the initial to the final complex structure are not noticed. Hence, the PC represents a suitable binding site for the lipid.

**Figure 5:**
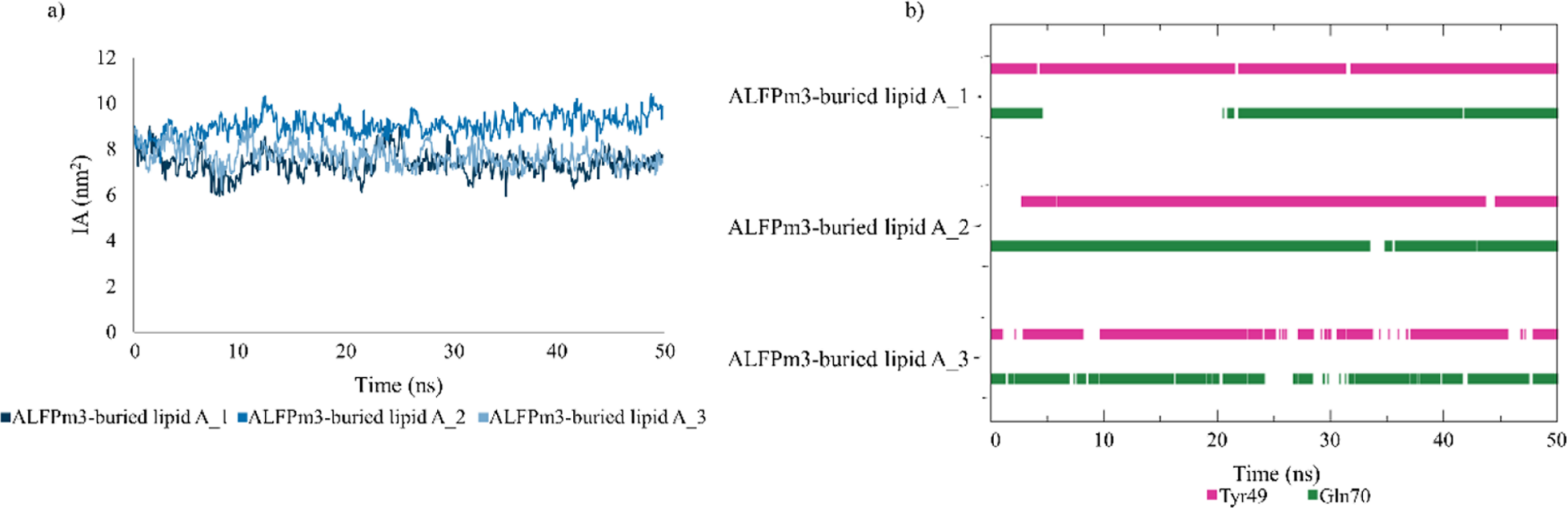
(a) IA of the AL3–LA complex and (b) occurrence of hydrogen bonds between AL3 and LA in the AL3–buried LA simulations.

The proposed binding site in the protein cleft is supported by the stable IA of the AL3–LA complex. Thereby, the IA remains stable around 8 nm^2^ during the simulations, as shown in Figure 5a. Additionally, the long-lived hydrogen bonds that LA establishes with amino acids located in the AL3 cavity also reveal the stable binding in that part of the protein. Thereby, the residues (Tyr49 and Gln70) that are involved in long-lived hydrogen bonds with the lipid in all AL3–buried LA simulations can be noticed in Figure 5b. Particularly, the percentage of occurrence of these hydrogen bonds is higher than 69% (Tyr49) and 67% (Gln70) in the three replicas. Therefore, it can be considered that these amino acids are key for the AL3–LA binding. Conversely, as discussed in Figure 3, stable salt bridges are not established between AL3 and LA when they bind in the protein cleft.

Regarding the strength of the AL3–LA binding when the lipid is inserted in the PC, it can be easily noticed from Table 2 that the binding free energy (Δ*G*_bind_) is more favorable for the AL3–buried LA simulations than for the AL3–LA simulations where the lipid does not completely reach the PC (i.e., first, second, and fourth replicas). Additionally, a similar binding free energy is obtained in the third replica of the AL3–LA simulations and in the AL3–buried LA simulations, which is reasonable since in all these simulations the lipid remains anchored in the protein’s cleft. Particularly, such enhanced binding strength of AL3 and LA arises from the more favorable van der Waals contribution to the binding free energy (Δ*G*_bind_^vdw^), as it was previously rationalized for the AL3–LA simulations. This observation emphasizes the importance of the hydrophobic interactions between the lipid aliphatic tails and hydrophobic amino acids of the PC so that AL3–LA bind tightly. On the other hand, the favorable binding free energies that are derived for the AL3–buried LA simulations yield an average binding constant of *K*_bind_ = 7 × 10^3^ M^–1^, thus demonstrating the strength of the AL3-LA binding.

**Table 2:** AL3–LA Binding Free Energies in the AL3–Buried LA Simulations

	Replica 1	Replica 2	Replica 3
Δ*G*_bind_^vdw^ (kJ·mol^–1^)	–19 ± 1	–27 ± 1	–25 ± 1
Δ*G*_bind_^elec^ (kJ·mol^–1^)	1 ± 1	2 ± 1	2 ± 1
Δ*G*_bind_ (kJ·mol^–1^)	–18 ± 1	–26 ± 1	–23 ± 1

Collectively, it can be concluded that the location of the LA binding site in the cavity of AL3 is supported by (i) the stable IA of the AL3-LA complex when the lipid is inserted in that cavity, (ii) the high occurrence of hydrogen bonds between LA and residues located in the protein cleft, and (iii) the tight AL3–LA binding. However, significant conformational changes of the protein structure are required so that AL3 could accommodate the lipid in its cavity, which may hamper that AL3 recovers its original conformation upon LA removal from the cleft.

The location of the LA binding site in the cavity of AL3 was also assessed by analyzing the recovery of the protein structure when the lipid is removed from its cavity due to the reversibility of their binding equilibrium. More specifically, the unfolding of AL3 as a result of the lipid removal from the PC implies that the binding site is not located in that part of the protein, since the reversibility of the protein-lipid interaction entails the recovery of the protein’s original conformation upon ligand removal. To assess the reversibility of the AL3–LA binding despite the opening of the PC to accommodate the lipid, we ran simulations in a 150 mM NaCl buffer of the apo-protein using as the initial structure one in which AL3 has an open conformation. These simulations will be referred to as “open apo-AL3 simulations” (see Table S1 for further details about the simulations’ nomenclature). The initial protein structure for these simulations was obtained from the AL3–buried LA simulations, as explained in the Methods section of the Supporting Information.

**Figure 6:**
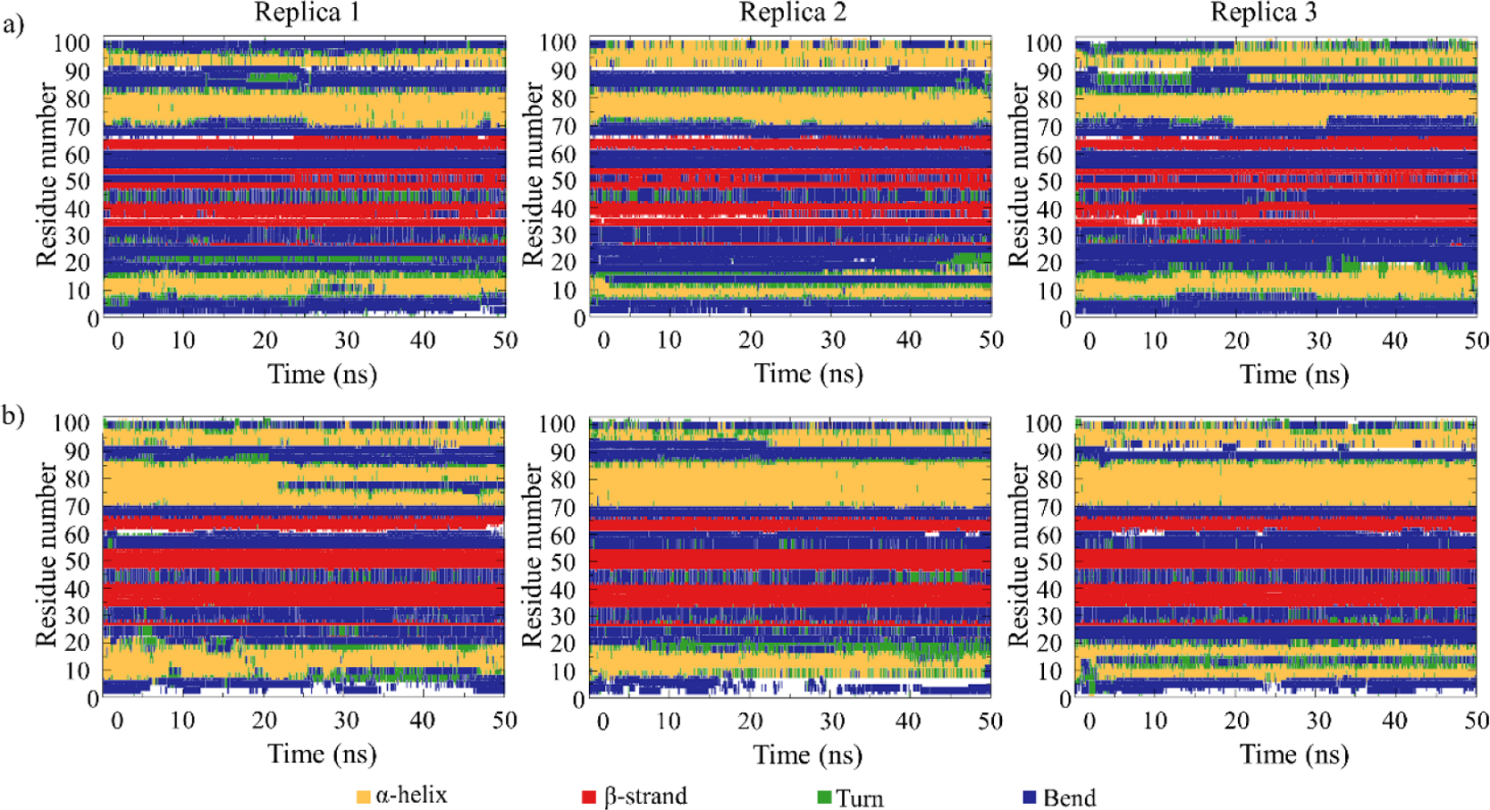
Secondary structure of AL3 in the (a) open apo-AL3 and (b) apo-AL3 simulations for the three replicas.

To examine the maintenance of the protein structure and thus verify the reversibility of the AL3–LA binding in the PC, the secondary structure of AL3 in these simulations was computed using the dictionary of secondary structure of proteins (DSSP) program. The outcomes of the DSSP analysis illustrated in Figure 6 show that the structure of AL3 in the open apo-AL3 simulations resembles that of the protein in the apo-AL3 simulations and in the initial structure derived by Yang et al.^31^ Specifically, it comprises three α-helices and four β-strands (depicted in yellow and red, respectively).

To sum up, the stability of the LA binding in the AL3 cleft and the reversibility of the AL3–LA binding have been verified in silico. Therefore, the location of the lipid binding site in the protein cleft has been further evidenced.

**Figure 7:**
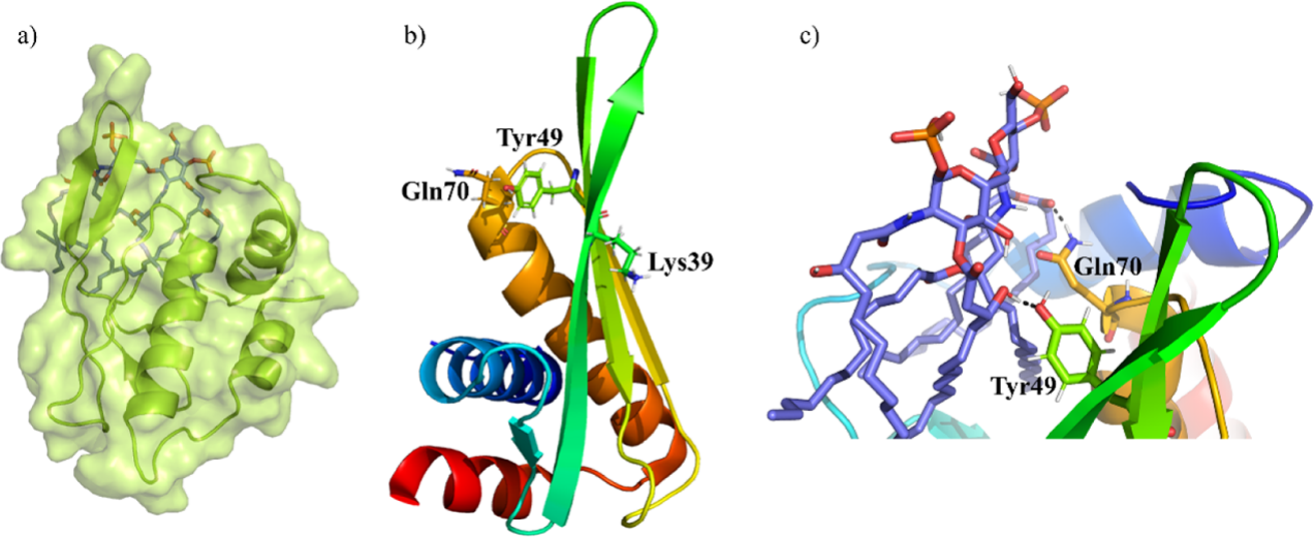
(a) AL3–LA binding pose derived from the MD simulations (surface and cartoon representation of AL3 in lime and stick representation of LA in purple), (b) key amino acids of AL3 for the interaction with LA, and (c) zoom representation of AL3 residues and LA constituents that are involved in long-lived hydrogen bonds.

Collectively, the in silico results provided throughout this work reveal a possible location of the LA binding site in the AL3 cavity (Figure 7a) and that the binding pose involves the opening of the PC and the burial of the lipid acyl chains in such cavity. Additionally, we demonstrated that hydrophobic interactions dominate the stable AL3–LA binding, which seems reasonable due to the high hydrophobic nature of LA. However, several positively charged residues of AL3 interact with the lipid phosphates, as noticed from the MD simulations. On the basis that both electrostatic and hydrophobic interactions are involved in the interaction of LPS with other molecules, such as MD2, FhuA, polymyxin B, or lysozyme,^10,42^ it could be suggested that the AL3–LA interaction mechanism consists of a two-stage process. First, the phosphate groups of LA are recognized by positively charged residues of AL3 through electrostatic interactions. Subsequently, the lipid movement toward the back side of the AL3 β-hairpin, where it buries its aliphatic tails in the protein cleft, is driven by hydrophobic interactions. Thus, a stable binding is achieved with the phosphates exposed to the medium and the acyl chains inserted in the PC. Three residues have been identified to be key in this interaction mechanism (Figure 7b). One of them, Lys39, is positively charged and located at the protein surface; it forms stable salt bridges with either of the lipid phosphates. Additionally, Tyr49 and Gln70, which are polar and neutral residues, belong to the PC and bind to the lipid through hydrogen bonds (Figure 7c). Two of these residues, viz., Lys39 and Tyr49, are conserved in LALF, as noticed from the sequence alignment included in Figure S5. The fact that two of the AL3 residues that were in silico predicted to interact with LA are conserved in LALF reinforces their crucial role in the interaction with LA and broadens the findings of this study to other ALFs.

### 2.3 In Vitro Validation of In Silico Predictions

The comprehensive determination of the AL3–LA interaction mechanism and binding mode requires that the in silico findings match what happens experimentally. Therefore, the importance of the AL3 residues identified by MD simulations and conserved in LALF (Lys39 and Tyr49) for interacting with LA was experimentally assessed. For that purpose, the variation in the binding ability after substituting these amino acids with others with opposite charge or polarity was quantified. Accordingly, the loss of the AL3’s ability to bind the lipid when the predicted amino acids are substituted would reveal their crucial role for interacting with LA. Hence, this methodology requires determining the binding ability of both the original and substituted AL3 toward LA to decipher the effect of the amino acid replacement.

Recently, the ability of a recombinant LALF protein to bind LPS has been experimentally determined by our research group.^27^ In the present work, we take advantage of that study and use the LALF protein instead of AL3 for the in vitro validation of the in silico findings. This choice stems from the similar interaction mechanism of LALF and AL3 with LPS that is expected due to the similarity of their 3D structures (see Section S1 of the Supporting Information) and the conservation of the AL3 residues that were identified as being key for interacting with LA in LALF. Thus, amino acid substitution has been performed on the DNA of LALF, and the ability to capture LPS of the substituted LALF has been compared to that of the wild-type LALF reported by our group (hereafter WT-LALF). Readers can refer to ref (27) for details about the synthesis and structure of WT-LALF. It is worth mentioning that Lys39 and Tyr49 of AL3 correspond to Lys37 and Tyr47 of WT-LALF, respectively. Therefore, Lys37 and Tyr47 are the amino acids to be substituted in WT-LALF in order to assess their role in the interaction of WT-LALF with LPS. To demonstrate the importance of Lys37, which is positively charged, in such interaction, it was substituted by Glu, which is negatively charged and the length of its side chain is similar to that of Lys; additionally, Tyr47, which is a polar and neutral residue, was substituted by Phe, which is nonpolar and has an aromatic ring as Tyr. It should be pointed out that these amino acids were individually replaced so that the role of each of them in the interaction of LALF with LPS could be independently assessed. Hence, two DNAs, one for each amino acid substitution (i.e., K37E and Y47F substitutions), were obtained by SDM, as described in the Methods section of the Supporting Information.

K37E-LALF and Y47F-LALF proteins were successfully overexpressed, as can be noticed from the appearance of a marked band at a molecular size of ∼58 kDa in the sodium dodecyl sulfate-polyacrylamide gel electrophoresis (SDS-PAGE) gel (Figure S6a). However, only the K37E-LALF protein could be purified, as noticed from the greatly marked bands at ∼58 kDa on the SDS-PAGE gel for K37E-LALF, which contrasts to the barely marked bands on the SDS-PAGE gel for Y47F-LALF (Figure S6b). The impossibility of purifying the Y47F-LALF protein may arise from the burial of the protein histidine tail during the synthesis, which hampers its access to the Ni^2+^ ions of the purification column and thus the protein purification through immobilized metal affinity chromatography. Since Y47F-LALF cannot be purified, the effect of such amino acid substitution on the ability of the protein to bind LPS has not been analyzed. Therefore, the LPS binding assays were only performed with K37E-LALF. These assays comprise the agarose beads functionalization with the K37E-LALF protein and their subsequent contact with LPS.

To analyze the ability to bind LPS of K37E-LALF and WT-LALF at different protein/LPS ratios (ϕ_protein/LPS_), two beads’ batches were functionalized. The functionalization of the agarose beads was monitored by measuring the protein concentration in the supernatant of the liquid phase, as described in the Methods section of the Supporting Information, which has been depicted in Figure S7. It can be easily noticed that the protein concentration in the supernatant decreases with time from the concentration of the protein solution contacted with the first and second batches of beads (4.40 and 2.87 mg·mL^–1^, respectively). This is due to the fact that the protein has been captured by the beads through the interaction of its histidine tail with the Ni^2+^ ions immobilized on the beads surface. The completion of the beads functionalization, which implies that the beads are no longer able to continue capturing K37E-LALF, was recognized by the reach of the protein-bead equilibrium.

Once the beads were functionalized, they were incubated with fluorescein isothiocyanate (FITC)-labeled *E. coli* O111:B4 LPS (FITC-LPS) in order to assess the binding ability of K37E-LALF by fluorescence techniques. The results of these contacts, as well as those of the WT-LALF/FITC-LPS contact,^27^ are included in Table 3. Two ϕ_protein/LPS_ values and contact times were tested. Particularly, for a protein/LPS ratio of around 300 and a contact time of 20 min, K37E-LALF functionalized beads are able to capture 26% LPS. Both proteins obtained a similar performance with ϕ_protein/LPS_ values 1 order of magnitude lower for WT-LALF than for K37E-LALF, concluding the worse LPS capture ability of K37E-LALF. In other words, when the same LPS mass is contacted with both proteins, the mass of K37E-LALF must be 1 order of magnitude higher than that of WT-LALF for obtaining a similar endotoxin capture. Hence, for WT-LALF/FITC-LPS ratios around 35.6 and a contact time of 20 min, the percentage of LPS capture is ∼30%. Moreover, when comparable protein/LPS concentration ratios (ϕ_protein/LPS_ ∼ 400) are accomplished for both K37E-LALF and WT-LALF, the percentage of LPS capture using beads functionalized with K37E-LALF is approximately half that when WT-LALF functionalized beads are used. Specifically, 85% LPS can be captured by WT-LALF functionalized beads in 10 min, whereas K37E-LALF functionalized beads are only able to capture 42% LPS despite increasing the contact time to 60 min. To sum up, the considerable reduction of the protein’s ability to bind LPS that has been noticed when Lys37 is substituted by Glu in WT-LALF demonstrates the key role of Lys37 in the interaction with LPS; thus, the in silico prediction is verified.

**Table 3:** Ability of K37E-LALF and WT-LALF to Sequestrate LPS

	ϕ_protein/LPS_	contact time (min)	LPS capture (%)	refs
K37E-LALF	∼300	20	26	this work
	∼400	60	42	
WT-LALF	35.6	20	∼30	(27)
	∼400	10	∼85	

## 3 Conclusions

Unraveling the interactions and the binding mode of LPS-sequestrating molecules and endotoxins is of paramount importance for moving forward on the design of therapeutics for effectively treating sepsis. In this regard, we have herein elucidated, for the first time to the best of our knowledge, a stable AL3–LA binding pose using MD simulations. Particularly, we have found that the LA binding site of AL3 is located in the hydrophobic cavity of the protein, and that the binding pose involves the burial of the lipid aliphatic tails in such cleft whereas the phosphate groups are exposed to the medium. This binding pose is consistent with that of LA and MD2 (a protein that also has a hydrophobic cavity). We also examined the thermodynamics governing the AL3–LA interaction and ascertained that their binding is mainly driven by hydrophobic interactions. Additionally, the importance of Lys39 and Tyr49 for the AL3–LA interaction has been identified. On the basis of the in silico results, we proposed a possible interaction mechanism for AL3 and LA, which entails the initial recognition of the lipid by the positively charged residues of AL3 (such as Lys39) and subsequently the stable binding in the protein cleft where Tyr49 plays a pivotal role. While the in vitro validation of the MD-derived results demonstrated that Lys39 is crucial for the AL3–LA interaction, the burial of the histidine tail in Y47F-LALF prevented the experimental assessment of the Tyr49 role in the binding process. Collectively, the insights gained in this work could prove valuable to go further on the rational design of LPS-sequestrating molecules, which are the cornerstone for the successful LPS removal in extracorporeal blood detoxification systems.

## 4 Methods

### 4.1 In Silico Methods

System construction, trajectory analysis, and sequence alignment are provided in the Supporting Information. All MD simulations were performed using the GROMOS11^43^ simulation package on NVIDIA graphics processing units. The GROMOS 54A8 force field^44^ was used to parameterize AL3, whereas LA was parameterized according to the GROMOS 53a6glyc parameter set^45^ with the phosphate groups taken from Margreitter and Oostenbrink^46^. The simple point charge (SPC) water model was used to solvate the systems in periodic rectangular boxes with a minimum solute-to-wall distance of 1.2 or 1.5 nm depending on the system. The systems were energy minimized using the steepest descent algorithm with a maximum of 3000 steps. Subsequently, Na^+^ and Cl^–^ ions were added to mimic the physiological conditions (i.e., NaCl concentration around 150 mM) and to neutralize the system. Thereafter, the equilibration of the systems was performed at 60 K with initial random velocities generated from a Maxwell–Boltzmann distribution; then, the systems were heated up to 300 K in five discrete steps, while simultaneously reducing the force constant for position restraints applied to the solute atoms from 2.5 × 10^4^ to 0 kJ·mol^–1^ nm^–2^. The production simulations were carried out at a constant temperature of 300 K and a constant pressure of 1 atm by using a weak coupling scheme with coupling times of 0.1 and 0.5 ps, respectively, and an isothermal compressibility of 4.575 × 10^–4^ kJ^–1^·mol·nm^3^. The leapfrog scheme was used to integrate Newton’s equations of motion with a time step of 2 fs. The SHAKE algorithm was applied to constrain the bond lengths of solute and solvent to their optimal values. Nonbonded interactions were computed using a twin-range cutoff scheme. More specifically, interactions up to a cutoff of 0.8 nm were evaluated at every time step from a pair-list that was updated every 10 fs. Between 0.8 and 1.4 nm, nonbonded interactions were calculated at pair-list updates and kept constant between the updates. For the long-range electrostatic interactions, a reaction-field contribution with a dielectric permittivity of 61^47^ outside the cutoff of 1.4 nm was added. In the systems where nuclear Overhauser effect (NOE) distance restraints were applied to the AL3 protein in the production simulations (see Section S3.1 of the Supporting Information), the force constant for distance restraining and the coupling time were set to 1000 kJ·mol^–1^·nm^–1^ and 1 ps, respectively. To ensure scrutiny of the reproducibility of MD results, three or four independent MD simulations of 50 ns length were performed for each system (see Table S1); specifically, these simulations solely differ in the initial velocity distribution.

### 4.2 In Vitro Methods

The materials and methods described in the following subsections refer to those related exclusively to the experimental contact of LPS and K37E-LALF. The details and procedures for obtaining the mutated proteins (namely, SDM, and protein overexpression, purification, and concentration) and for the functionalization of agarose beads with the K37E-LALF protein are included in the Supporting Information.

## 5 Materials

Agarose beads were obtained from GE Healthcare, and FITC-LPS was purchased from Merck. FITC-LPS solutions were prepared with Milli-Q water, and the FITC-LPS concentration was measured using the SPARK multimode microplate reader (Tecan) with multiwell cell culture plates (96 wells) that were acquired from VWR.

## 6 Experimental Procedure

To assess the ability of K37E-LALF to sequestrate LPS, beads functionalized with this protein were contacted with FITC-LPS, and the LPS removal from the solution was quantified. Following the procedure of Basauri et al.,^27^ different K37E-LALF/FITC-LPS ratios (ϕ_protein/LPS_) were tested in order to prove the worsening of the LALF’s ability to sequestrate LPS when it is mutated, even when outstandingly favorable conditions for the binding (protein mass significantly higher than LPS mass) were used. Specifically, 75 μL of a FITC-LPS solution with a concentration of 200 or 250 μg·mL^–1^ were incubated with the functionalized beads under gentle shaking during different times. Afterward, the FITC-LPS/beads mixture was centrifuged, and samples of the supernatant liquid were pipetted into a 96 well plate in order to measure the LPS concentration in the supernatant by fluorescence techniques, since LPS contained fluorescent conjugates, and thus LPS concentration correlated with the intensity of fluorescence. Measurements were carried out in a Spark multimode microplate reader using excitation/emission wavelengths of 495 and 525 nm, respectively.

## Supporting Information Available

Descriptions of anti-lipopolysaccharide factors and Gram-negative bacterial lipid A; in silico and in vitro methods; sequence alignment; MD and experimental results (PDF)

## Notes

The GROMOS11 simulation package was used to perform all MD simulations (https://www.gromos.net/). MD trajectories were analyzed with the GROMOS++ analysis tools (https://www.gromos.net/) and in-house scripts, which are made available upon request. The commercial software Molecular Operating Environment (MOE) (https://www.chemcomp.com/Products.htm) was used to modify the LA structure. Figures were created with Grace (https://plasma-gate.weizmann.ac.il/Grace/), licensed PyMOL (https://pymol.org/2/), and the ChemDraw online server (https://chemdrawdirect.perkinelmer.cloud/js/sample/index.html). Atomic coordinates for AL3 and LA are available in the PDB (https://www.rcsb.org/) under the accession codes 2JOB and 1QFG, respectively. The data generated in this study are available in the article and its Supporting Information.
