# Supplementary material for: Insights into the Binding Mode of Lipid A to the Anti-lipopolysaccharide Factor ALFPm3 from Penaeus monodon: An In Silico Study through MD Simulations
Source: J Chem Inf Model. 2023 Apr 7;63(8):2495–504. doi: 10.1021/acs.jcim.3c00173 (PMC10131219; doi:10.1021/acs.jcim.3c00173)
Supplement: Supplementary file 1 — ci3c00173_si_001.pdf [file ci3c00173_si_001.pdf]

Supporting Information

Insights into the Binding Mode of Lipid A to the Anti-lipopolysaccharide Factor ALFPm3 from *Penaeus monodon*: An *in Silico* Study through MD Simulations

Cristina González-Fernández[1], *Christoph Öhlknecht*[2], *Matthias Diem*[2], *Yerko Escalona*[2], *Eugenio Bringas*[1], *Gabriel Moncalián*[3], *Chris Oostenbrink*[2] *and Inmaculada Ortiz*[1]*

[1] Departamento de Ingenierías Química y Biomolecular, Universidad de Cantabria, Avda. Los Castros, s/n, 39005 Santander, Spain

[2] Institute for Molecular Modeling and Simulation, BOKU – University of Natural Resources and Life Sciences, Muthgasse 18, 1190, Vienna, Austria

[3] Departamento de Biología Molecular, Universidad de Cantabria and Instituto de Biomedicina y Biotecnología de Cantabria (IBBTEC), Universidad de Cantabria-CSIC, 39011 Santander, Spain

***** Correspondence ortizi@unican.es; Tel.: +34-94-220-1585

1. DESCRIPTION OF ANTI-LIPOPOLYSACCHARIDE FACTORS

Anti-lipopolysaccharide factors (ALFs) are small basic proteins of around 100 amino acids with a hydrophobic N-terminal region and two conserved cysteine residues. The ALF isoform 3 from *Penaeus monodon* (ALFPm3), which has been referred to as "AL3" for the sake of simplicity and the *Limulus polyphemus* ALF (known as LALF) have an extremely similar three-dimensional (3D) structure. Particularly, the LALF and AL3 3D structures, which have been solved by X-ray crystallography and nuclear magnetic resonance (NMR), respectively, comprise three α-helices packed against a four-stranded β-sheet.[1–6] As detailed in the works of Hoess et al.[7] and Yang et al.[5], who determined the structures of LALF and AL3, respectively, both proteins bear a central β-hairpin that is constrained by a disulfide bond formed by two conserved cysteine residues. The structure of AL3 has been depicted in Figure S1, since in contrast to that of LALF, it is deposited in the Protein Data Bank (PDB) under the accession code 2JOB.

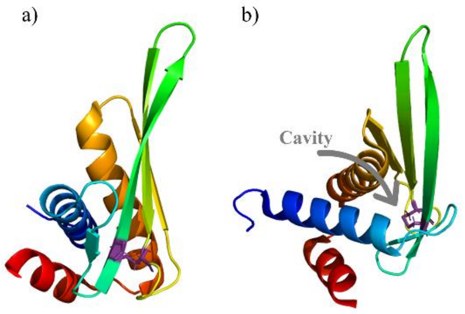

Figure S1. Cartoon model representation of AL3 showing (a) the front and lateral sides, and (b) the protein cavity. AL3 is rainbow colored from N-terminus (blue) to C-terminus (red); cysteine residues are represented in stick model and colored in purple.

2. DESCRIPTION OF GRAM-NEGATIVE BACTERIAL LIPID A

Escherichia coli lipid A (LA), whose structure has been represented in Figure S2, has been considered in this study to elucidate its interaction with AL3. It comprises a β-

(1→6)-linked glucosamine disaccharide backbone that is phosphorylated at positions 1 and 4' of the glucosamines GlcN I and GlcN II, respectively.[8,9] Additionally, this glucosamine disaccharide is acylated at positions 2 and 3 of both GlcN residues. Specifically, the primary fatty acids consist of (*R*)-3-hydroxymyristoyl groups. In GlcN II these primary acyl chains are esterified with two secondary acyl chains, namely, lauroyl group and myristoyl group via amide and ester linkage, respectively.[8–10]

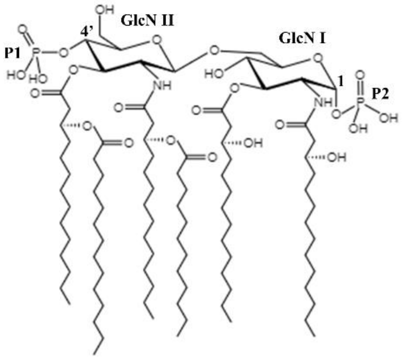

Figure S2. Structure of *E. coli* LA (representation drawn using the ChemDraw online server).

3. METHODS

3.1. *In silico* methods

To elucidate the AL3-LA interaction mechanism and binding mode, molecular dynamics (MD) simulations of AL3 in absence (apo- AL3) and presence of LA (AL3-LA) were performed. Additionally, MD simulations of both the AL3-LA complex where the lipid is buried in the protein cleft (AL3-buried LA) and apo- AL3 after AL3-buried LA simulations (open apo- AL3) were also carried out. A summary of the simulations that have been performed in this study has been included in Table S1. It is important to note that in the open-apo AL3 simulations nuclear Overhauser effect (NOE) distance restraints are applied to facilitate the recovery of the original AL3 structure from its open state; otherwise, these simulations would be highly computationally demanding due to the great

conformational change of the protein that is required to be sampled. These restraints were derived from the NMR restraints for the AL3 structure that are available in the PDB and applied in the form of half-harmonic attractive distance restraints to ensure that the experimentally observed upper bounds to interproton distances were obeyed.

Table S1. Summary of the MD simulations.

System denomination	LA position respect to AL3	NOE distance restraints	Replicas x simulation time (ns)
**Apo- AL3**	-	NO	3 x 50
**AL3-LA**	Facing the external side of the protein β-hairpin	NO	4 x 50
**AL3-buried LA**	Buried in protein cleft	NO	3 x 50
**Open apo- AL3**	-	YES	3 x 50

System construction

The NMR structure of apo- AL3 was retrieved from the PDB (accession code: 2JOB). Particularly, from the 15 conformers of the NMR structural ensemble that are available, the first one was used since it was the best representative conformer as stated by Yang et al.[5] The protonation states were assigned considering pH equal to 7.0; the protonation state of the histidines was considered to be neutral, being the proton located on N_δ. The coordinates for LA were taken from the work of Ferguson et al.[9] To build the AL3-LA bound structure for the AL3-LA simulations (see Table S1), we considered as the

cornerstone the hypothesis of Yang et al.[5] which states that a similar LA binding site is shared by LPS binding proteins. Hence, we followed a similar procedure as the one Yang et al.[5] used to propose the AL3 amino acids that could belong to the LA binding site. Thereby, from the structures of the FhuA-LPS complex (PDB ID: 1QFG) and AL3, we superimposed, using PyMOL[11], the amino acids of these proteins that were found to be counterparts according to Yang et al.[5] Particularly, the residues Glu304, Lys306, Lys351, Arg382, Arg384, Lys439 and Lys441 of FhuA were superimposed with Glu25, Lys26, Lys35, Lys39, Arg52 and Lys50 of AL3, respectively; an optimum match of 0.87 Å was obtained from that superimposition. Subsequently, the FhuA structure and the constituents of LPS different from LA were removed. Finally, we modified the LA structure using the Molecular Operating Environment (MOE) software[12] so that the typical structure of *E. coli* LA could be derived. As a result, the AL3-LA complex to be used as initial structure for the MD simulations was obtained. On the other hand, the structure for starting the simulations where LA is buried in the protein cleft (AL3-buried LA simulations, see Table S1) was derived by extracting a structure of the AL3-LA complex with the most favorable van der Waals energy from the third replica of the AL3-LA simulations. Finally, the initial structure for the apo- AL3 simulations after LA binding in the protein core (open apo- AL3 simulations, see Table S1) was obtained by pulling out a structure of the AL3-LA complex where the lipid is considerably inserted in the protein cavity from the AL3-buried LA simulations and removing the LA atoms.

Trajectory analysis

Time series analysis of the simulated trajectories was performed using the GROMOS++[13] analysis tools and in-house scripts. Thereby, the dssp and rmsd programs of GROMOS++ were used to compute the secondary structure content of AL3 according to the Dictionary of Secondary Structures of Proteins (DSSP) and the atom-positional root-mean-square

deviation (RMSD) of its backbone atoms (C-α, N, C) with respect to the AL3 minimized structures, respectively. The GROMOS++ program sasa was also used to derive the solvent accessible surface area (SASA) of AL3, LA and AL3-LA complex. On the basis that the interface area (IA) of the AL3-LA complex is not accessible to the solvent, it can be computed from the SASA of the protein ($SASA_{protein}$), the lipid ($SASA_{lipid}$) and the protein-lipid complex ($SASA_{complex}$) as follows:

$$IA = \frac{SASA_{protein} + SASA_{lipid} - SASA_{complex}}{2} \qquad \text{(Eq. S1)}$$

Additionally, the clustering analysis was performed using the GROMOS++ programs: rmsdmat, cluster and postcluster. The conformational clustering was based on the pairwise RMSD between configurations, considering a cutoff of 0.6 nm to identify similar structures.

The hydrogen bonds and salt bridges between AL3 and LA were identified from geometric criteria. Hydrogen bonds are considered to be formed if the hydrogen-acceptor distance and the donor-hydrogen-acceptor angle are smaller than 0.25 nm and higher than 135°, respectively. Similarly, to recognize the salt bridges between the central phosphorous atoms of LA and the CZ, NZ, CG or CE1 of the arginine, lysine, glutamic acid or histidine residues of AL3 respectively, a cutoff distance of 0.7 nm was considered. This broad cutoff was selected on the basis of the study of Garate and Oostenbrink[14], who also used this cutoff for computing the salt bridges between LA other biomolecules.

The binding free energy (ΔG_{bind}) was determined using the linear interaction energy (LIE) method, which only requires the end-states simulations[14], according to Eq. S2:

$$\Delta G_{bind} = \alpha \Delta \langle U_{l-s}^{vdW} \rangle + \beta \Delta \langle U_{l-s}^{elec} \rangle \qquad \text{(Eq. S2)}$$

Where $\langle U_{l-s}^{vdW} \rangle$ and $\langle U_{l-s}^{elec} \rangle$ represent the MD energy averages of the nonbonded van der Waals and electrostatic interactions, respectively, between the ligand (LA) and its surrounding environment, i.e., the receptor (AL3) binding site or the solvent.[15–17] The empirical parameters α=0.18 and β=0.09 were used, as reported in the literature for calculating the binding free energy of LA with other biomolecules.[14,18,19] It is worth mentioning that the first two nanoseconds were discarded for calculating ΔG_{bind}, so that it could be computed from the equilibrated part of the simulation.

Once ΔG_{bind} is computed, the equilibrium binding constant can be calculated using the following equation:

$$\Delta G_{bind} = -R\, T \ln K_{binding} \qquad \text{(Eq. S3)}$$

where R is the ideal gas constant and T the absolute temperature.

Regarding the generation of the graphics included throughout this work, PyMOL[11] was employed to derive the molecular graphic figures. Additionally, data from calculations of MD trajectories were plotted using the Grace (GRaphing, Advanced Computation and Exploration of data) tool.

Sequence alignment

The amino acid sequences of different ALFs were aligned using the multiple sequence alignment program Clustal Omega web server from the European Bioinformatics Institute (EBI) using default settings.[20]

3.2. *In vitro* methods

The materials and methods herein detailed refer to those related to: (i) the obtention of the mutated proteins, namely K37E-LALF and Y47F-LALF, and (ii) the functionalization of agarose beads with the K37E-LALF protein. The procedure for obtaining these

proteins involves: site-directed mutagenesis (SDM) to obtain the DNAs that code the different amino acid substitution, and protein overexpression, purification and concentration.

Materials

Primers were designed and purchased to StabVida. The QuikChange II SDM kit was obtained from Agilent Technologies, Inc. Antibiotics, i.e., kanamycin (Kn) and gentamycin (Gn) were from Apollo Scientific. Isopropyl β-D-1-thiogalactopyranoside (IPTG) and PageRuler^{TM} Plus Prestained Protein Ladder were purchased to Thermo Scientific. Phenylmethylsulfonyl fluoride (PMSF), lysozyme from chicken egg white, and imidazole were form Sigma Aldrich. Dithiothreitol (DTT), sodium dodecyl sulfate (SDS), and the GeneJet plasmid miniprep kit were obtained from Fisher Scientific, and BlueSafe protein stain to Nzytech. Tris-HCl, NaCl, Luria Bertani (LB) medium and LB agar medium were acquired from Scharlab, S. L. HisTrap HP histidine-tagged protein purification columns (5 mL) were purchased to GE Healthcare. 30 kDa Centricon® centrifugal filters (Amicon® Ultra) were obtained from Merck Millipore. Agarose beads were obtained from GE Healthcare.

C1000 TouchTM Thermal Cycler (Biorad) was used for the polymerase chain reactions (PCR). Optical density (OD), DNA and protein concentration measurements were carried out in a Nanodrop 2000c spectrophotometer (Thermo Scientific). A microPulser electroporator (Biorad) and 2 mm electroporation cuvettes (Molecular Bioproducts) were used for the transformation of mutated DNA. Avanti J-30I centrifuge (Beckman Coulter, USA), Sorvall WX Ultra Series Centrifuge (Thermo Scientific) and Centrifuge 5810R (Eppendorf) were used for different centrifugation requirements. Electrophoresis was performed using the mini-protean system (Biorad). A MiniPlus 3 peristaltic pump (Gilson) was used to equilibrate and load the HisTrap HP histidine-tagged protein

purification columns, and the fast protein liquid chromatography (FPLC) system ÄKTA type (GE Healthcare) for target protein elution from these columns. Protein concentration was measured in a Nanodrop 2000c spectrophotometer (Thermo Scientific).

Experimental procedure

LALF SDM was performed using the QuikChange II SDM kit with *PfuUltra* high-fidelity DNA polymerase following the manufacturer's instructions. Thereby, the thermocycling conditions for PCR established in the protocol were used. Then, the amplified DNAs (one with the K37E and the other with the Y47F substitutions) were transformed into XL1-Blue supercompetent cells, which were subsequently plated onto LB agar plates containing Kn and incubated overnight for plasmid DNA extraction using miniprep. Once the presence of the desired mutations was verified by sequencing, the plasmids were individually transformed into *E. coli* ArcticExpress competent cells by electroporation. Transformed cells were grown in LB medium (1L) containing Kn and Gn, at 37 °C with shaking, until an OD of 0.4-0.6 was reached. At this point, the expression of the K37E-LALF and Y47F-LALF proteins was induced with IPTG to a final concentration of 0.5 mM at 18 °C overnight. Subsequently, the cultures were centrifuged at 4,000 rpm and 4 °C for 20 minutes. The resulting pellets were frozen at -80 °C; samples of the pellets were taken prior to be freezing to verify protein expression by electrophoresis under denaturalizing conditions.

For the purification of the aforementioned proteins, the pellets were thawed and resuspended in 50 mL of lysis buffer (Table S2) supplemented with 250 $\mu g \cdot mL^{-1}$ of lysozyme and 300 µM PMSF and incubated during 30 minutes. Cell lysis was performed by sonicating the previous mixture during 3 cycles of 1 minute at intervals of 1 minute on ice. Then, the cell lysate was ultracentrifuged at 40,000 rpm for 30 minutes at 4 °C. Supernatants were loaded on a HisTrap HP histidine-tagged protein purification column,

that was previously equilibrated with buffer A (Table S2), and protein purification was carried out by immobilized metal affinity chromatography (IMAC). The K37E-LALF and Y47F-LALF proteins were eluted from the column by an imidazole concentration gradient between buffers A and B (Table S2). Samples of eluted fractions were analyzed by sodium dodecyl sulfate-polyacrylamide gel electrophoresis (SDS-PAGE) to confirm the presence of the band at ~58 kDa. Then, K37E-LALF containing fractions were concentrated and buffer exchanged to increase the concentration of the target protein while reducing the concentration of salts (i.e., NaCl, and imidazole). For that purpose, the product of the purification was mixed with buffer C (Table S2) in Centricons and centrifuged at 4,200 rpm and 4 °C.

Subsequently, agarose beads were functionalized with K37E-LALF. Firstly, 500 µl of agarose beads were centrifuged to remove the ethanol where they are initially suspended and resuspended in 500 µl of buffer D (see Table S2). Then, beads were rinsed for three times; in each rinse cycle, beads were centrifuged and resuspended in the previous buffer. Finally, beads were incubated with the mutated protein under gentle shaking; as a result, the beads become functionalized by that protein due to the strong affinity of the histidine tag tail of K37E-LALF to the Ni^{2+} ions present on the surface of the agarose beads. Beads functionalization was monitored from the initial protein concentration (in the absence of beads) and the protein concentration in the supernatant (in the presence of beads) at different times. Thereby, the difference between these protein concentrations (i.e., initial and in the supernatant) represents the amount of protein that is anchored on the beads surface. Once the bead-protein equilibrium was reached, which implies that beads were unable to continue capturing the protein, beads were washed 3 times and resuspended in buffer D following the same procedure as that above-mentioned in order to remove the unbound protein from the beads surface. Thus, protein concentration was also measured

in the washing solution to verify that K37E-LALF was successfully anchored to the beads. All mentioned protein concentrations were measured in a Nanodrop 2000c spectrophotometer at 280 nm, specifying the protein size (57.9 kDa) and the molar extinction coefficient (ε=104.5 mol·L^{-1}).

Table S2. Composition of the buffers that have been used for experimentally investigating the mutated LALF-LPS interaction.

Buffer	Composition
**Lysis buffer**	100 mM Tris-HCl, 500 mM NaCl, 1% Triton X-100, 5 mM DTT pH=7.5; supplemented with lysozyme (100 µg·mL^{-1}) and PMSF when used
**Buffer A**	100 mM Tris-HCl, 500 mM NaCl, 50 mM imidazole, 5 mM DTT pH= 7.5
**Buffer B**	100 mM Tris-HCl, 500 mM NaCl, 500 mM imidazole, 5 mM DTT pH= 7.5
**Buffer C**	50 mM Tris-HCl, 150 mM NaCl, 5 mM DTT, pH= 7.5
**Buffer D**	50 mM Tris-HCl, 150 mM NaCl, pH= 7.5

4. SIMULATIONS CONVERGENCE

The convergence of the MD simulations of all systems under study (i.e., AL3 in the apo and LA-bound states) was evaluated by computing the RMSD of the protein backbone atoms (C-α, N, C) with respect to its minimized AL3 structure. The time-series of these RMSDs have been depicted in Figure 2 of the manuscript and Figure S3 of the Supporting Information. As shown from these Figures, the RMSDs of all simulations appear to be mostly converged after 20 ns, which verifies the convergence of the simulations.

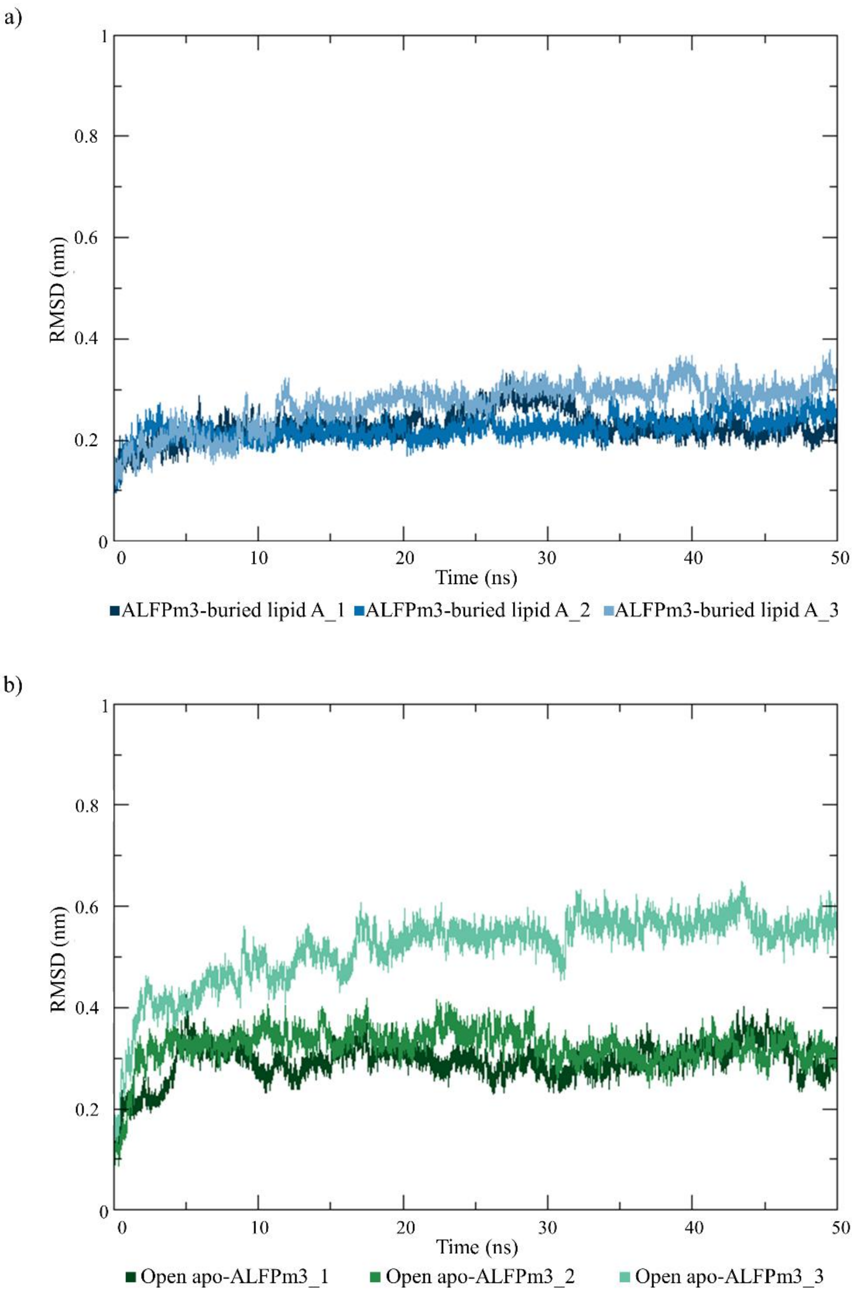

Figure S3. Time-series of the RMSD of the AL3 backbone atoms in the (a) AL3-buried LA, and (b) open apo- AL3 simulations.

5. CONFORMATIONAL CLUSTERING ANALYSIS

Clustering analysis was performed to identify similar AL3-LA conformations from the four replicas of the AL3-LA simulation (see Table S1). In Figure S4a, the number of structures that comprise the first three clusters are presented. It can be noticed that the first cluster contains structures from the four replicas of the AL3-LA simulation. This fact implies that similar conformations are shared by these four simulations. Specifically, the representative conformation (central member structure) of the first cluster, which is depicted in Figure S4b, features the lipid trying to reach the back side of the protein β-hairpin.

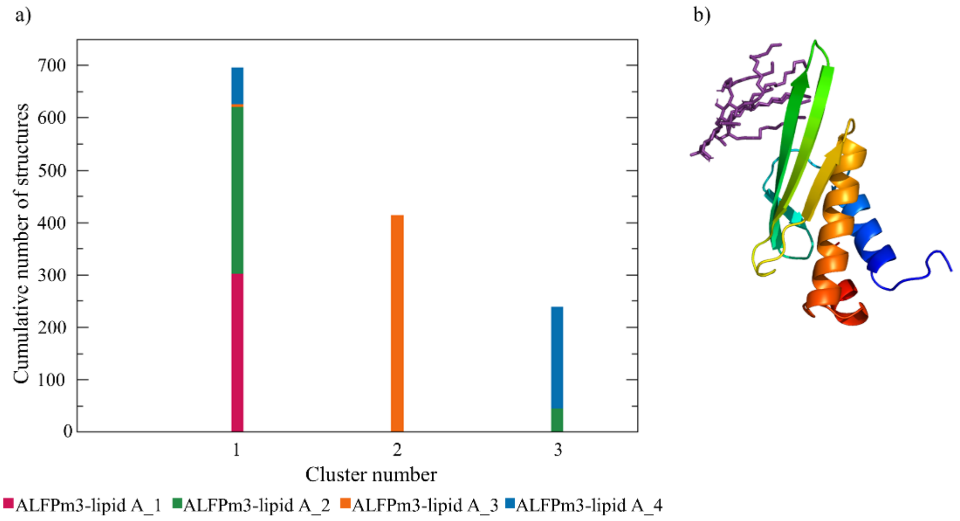

Figure S4. Conformational clustering analysis of the AL3-LA binding pose for the AL3-LA simulations: (a) number of configurations from each replica that constitutes the first three clusters, (b) central member structure of the first cluster.

6. BROADENING *IN SILICO* FINDINGS TO OTHER ALFs: SEQUENCE ALIGNMENT

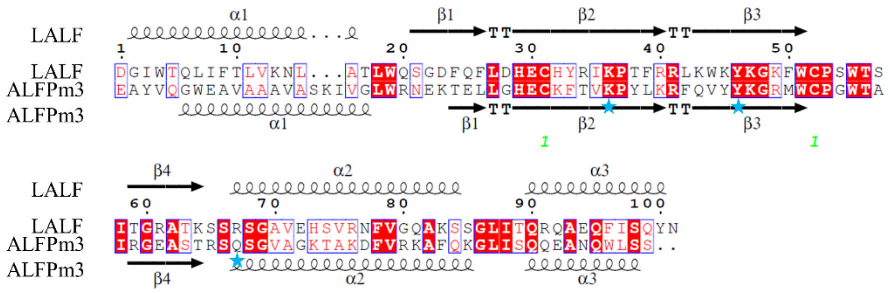

Figure S5. Alignment of AL3 and LALF amino acid sequences. Amino acids in the 39th, 49th and 70th positions of AL3 and their counterparts in LALF are indicated with blue stars.

7. *IN VITRO* VALIDATION OF *IN SILICO* PREDICTIONS

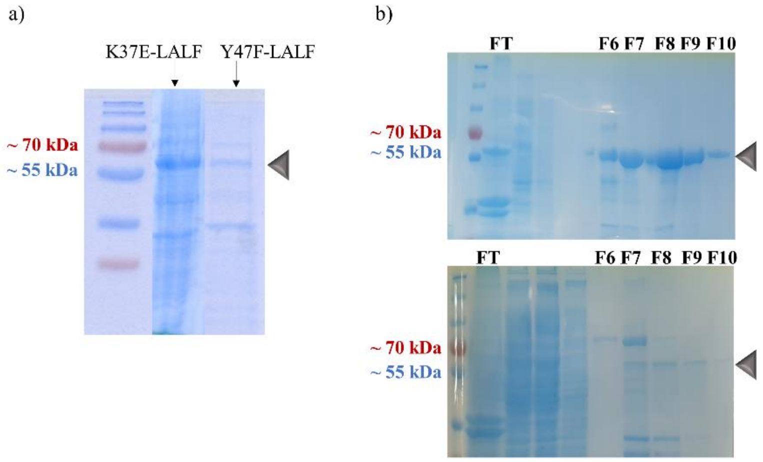

Figure S6. SDS-PAGE gels showing (a) the overexpression of K37E-LALF and Y47F-LALF, and (b) the purification fractions (FT: flowthrough; F6-F10: fractions from 6 to 10) of K37E-LALF (upper gel) and Y47F-LALF (lower gel). Page Ruler protein ladder sizes are indicated on the left of each SDS-PAGE gel.

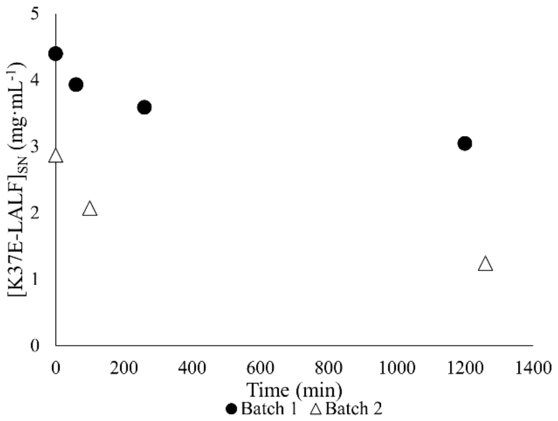

Figure S7. Evolution of the K37E-LALF concentration in the supernatant (SN) during the functionalization of agarose beads.

REFERENCES

(1) Jaree, P.; Tassanakajon, A.; Somboonwiwat, K. Effect of the Anti-Lipopolysaccharide Factor Isoform 3 (ALFPm3) from *Penaeus Monodon* on *Vibrio Harveyi* Cells. *Dev. Comp. Immunol.* **2012**, *38*, 554–560. https://doi.org/10.1016/j.dci.2012.09.001.

(2) Somboonwiwat, K.; Marcos, M.; Tassanakajon, A.; Klinbunga, S.; Aumelas, A.; Romestand, B.; Gueguen, Y.; Boze, H.; Moulin, G.; Bachère, E. Recombinant Expression and Anti-Microbial Activity of Anti- Lipopolysaccharide Factor (ALF) from the Black Tiger Shrimp *Penaeus Monodon*. *Dev. Comp. Immunol.* **2005**, *29*, 841–851. https://doi.org/10.1016/j.dci.2005.02.004.

(3) Rosa, R. D.; Vergnes, A.; de Lorgeril, J.; Goncalves, P.; Perazzolo, L. M.; Sauné, L.; Romestand, B.; Fievet, J.; Gueguen, Y.; Bachère, E.; Destoumieux-Garzón, D. Functional Divergence in Shrimp Anti-Lipopolysaccharide Factors (ALFs): From Recognition of Cell Wall Components to Antimicrobial Activity. *PLoS One* **2013**, *8*, e67937. https://doi.org/10.1371/journal.pone.0067937.

(4) Schmitt, P.; Rosa, R. D.; Destoumieux-Garzón, D. An Intimate Link between Antimicrobial Peptide Sequence Diversity and Binding to Essential Components of Bacterial Membranes. *Biochim. Biophys. Acta - Biomembr.* **2016**, *1858*, 958–970. https://doi.org/10.1016/j.bbamem.2015.10.011.

(5) Yang, Y.; Boze, H.; Chemardin, P.; Padilla, A.; Moulin, G.; Tassanakajon, A.; Pugnière, M.; Roquet, F.; Destoumieux-Garzón, D.; Gueguen, Y.; Bachère, E.; Aumelas, A. NMR Structure of rALF-Pm3, an Anti-Lipopolysaccharide Factor from Shrimp: Model of the Possible Lipid A-Binding Site. *Biopolymers* **2009**, *91*, 207–220. https://doi.org/10.1002/bip.21119.

(6) Tassanakajon, A.; Amparyup, P.; Somboonwiwat, K.; Supungul, P. Cationic Antimicrobial Peptides in Penaeid Shrimp. *Mar. Biotechnol.* **2011**, *13*, 639–657. https://doi.org/10.1007/s10126-011-9381-8.

(7) Hoess, A.; Watson, S.; Siber, G. R.; Liddington, R. Crystal Structure of an Endotoxin-Neutralizing Protein from the Horseshoe Crab, *Limulus* Anti-LPS Factor, at 1.5 A Resolution. *EMBO J.* **1993**, *12*, 3351–3356.

(8) Steimle, A.; Autenrieth, I. B.; Frick, J. S. Structure and Function: Lipid A Modifications in Commensals and Pathogens. *Int. J. Med. Microbiol.* **2016**, *306*, 290–301. https://doi.org/10.1016/j.ijmm.2016.03.001.

(9) Ferguson, A. D.; Welte, W.; Hofmann, E.; Lindner, B.; Holst, O.; Coulton, J. W.; Diederichs, K. A Conserved Structural Motif for Lipopolysaccharide Recognition by Procaryotic and Eucaryotic Proteins. *Structure* **2000**, *8*, 585–592. https://doi.org/10.1016/S0969-2126(00)00143-X.

(10) Knirel, Y. A. Valvano, M. A. *Bacterial Lipopolysaccharides. Structure, Chemical Synthesis, Biogenesis and Interactions with Host Cells*; Springer: New York, 2011.

(11) Schrödinger, LLC. The PyMOL Molecular Graphics System.

(12) Molecular Operating Environment (MOE); Chemical Computing Group ULC: 1010 Sherbooke St. West, Suite #910, Montreal, QC, Canada.

(13) Eichenberger, A. P.; Allison, J. R.; Dolenc, J.; Geerke, D. P.; Horta, B. A. C.; Meier, K.; Oostenbrink, C.; Schmid, N.; Steiner, D.; Wang, D.; van Gunsteren, W. F. GROMOS++ Software for the Analysis of Biomolecular Simulation Trajectories. *J. Chem. Theory Comput.* **2011**, *7*, 3379–3390. https://doi.org/10.1021/ct2003622.

(14) Garate, J. A.; Oostenbrink, C. Lipid A from Lipopolysaccharide Recognition: Structure, Dynamics and Cooperativity by Molecular Dynamics Simulations. *Proteins Struct. Funct. Bioinforma.* **2013**, *81*, 658–674. https://doi.org/10.1002/prot.24223.

(15) Ljungberg, K. B.; Marelius, J.; Musil, D.; Svensson, P.; Norden, B.; Åqvist, J. Computational Modelling of Inhibitor Binding to Human Thrombin. *Eur. J. Pharm. Sci.* **2001**, *12*, 441–446. https://doi.org/10.1016/S0928-0987(00)00185-8.

(16) Miranda, W. E.; Noskov, S. Y.; Valiente, P. A. Improving the LIE Method for Binding Free Energy Calculations of Protein-Ligand Complexes. *J. Chem. Inf. Model.* **2015**, *55*, 1867–1877. https://doi.org/10.1021/acs.jcim.5b00012.

(17) Gilson, M. K.; Zhou, H. X. Calculation of Protein-Ligand Binding Affinities. *Annu. Rev. Biophys. Biomol. Struct.* **2007**, *36*, 21–42. https://doi.org/10.1146/annurev.biophys.36.040306.132550.

(18) Garate, J. A.; Stöckl, J.; Fernández-Alonso, M. D. C.; Artner, D.; Haegman, M.; Oostenbrink, C.; Jiménez-Barbero, J.; Beyaert, R.; Heine, H.; Kosma, P.; Zamyatina, A. Anti-Endotoxic Activity and Structural Basis for Human MD-2·TLR4 Antagonism of Tetraacylated Lipid A Mimetics Based on βGlcN(1-1) αGlcN Scaffold. *Innate Immun.* **2015**, *21*, 490–503. https://doi.org/10.1177/1753425914550426.

(19) Borio, A.; Holgado, A.; Garate, J. A.; Beyaert, R.; Heine, H.; Zamyatina, A. Disaccharide-Based Anionic Amphiphiles as Potent Inhibitors of Lipopolysaccharide-Induced Inflammation. *ChemMedChem* **2018**, *13*, 2317–2331. https://doi.org/10.1002/cmdc.201800505.

(20) Sievers, F.; Wilm, A.; Dineen, D.; Gibson, T. J.; Karplus, K.; Li, W.; Lopez, R.;

McWilliam, H.; Remmert, M.; Söding, J.; Thompson, J. D.; Higgins, D. G. Fast, Scalable Generation of High-Quality Protein Multiple Sequence Alignments Using Clustal Omega. *Mol. Syst. Biol.* **2011**, *7*, 539. https://doi.org/10.1038/msb.2011.75.
